# Supplementary material for: Exploring the influence of historical storytelling on cultural heritage tourists’ revisit intention: A case study of the Mogao Grottoes in Dunhuang
Source: PLoS One. 2024 Sep 19;19(9):e0307869. doi: 10.1371/journal.pone.0307869 (PMC11412526; doi:10.1371/journal.pone.0307869)
Supplement: S1 Appendix — (DOCX) [file pone.0307869.s001.docx]

**Appendix**

**Questionnaire**

Dear ladies/gentlemen，

Greetings! Thank you very much for accepting our questionnaire in your busy schedule.

I am a master's student at the Northwest Normal University Tourism Institute, and I am doing research on the impact of historical narratives in heritage tourism on tourists' willingness to return. Your participation in this questionnaire is voluntary and your opinion is very important to us. This questionnaire is filled in anonymously, the content you fill in is only for academic research. We will strictly protect your personal information to ensure its security and confidentiality. There is no right or wrong answer to any question. Please don't spend too much time thinking about the answer to the question. We just need to know how you feel immediately. Please choose an answer to each of the following questions according to your own judgement. It will take you six minutes to complete this questionnaire. We appreciate your help very much.

**Part 1: Personal information**

Please mark your answers with a “√”:

## 1.Your gender：

⬜Male ⬜Female

## 2.Your age：

⬜Under 18 years old ⬜18-25 years old ⬜26-40 years old

⬜41-60 years old ⬜Over 60 years old

## 3.Your educational background：

⬜Junior high school and below ⬜Senior high school

⬜College degree ⬜Master degree and above

## 4.Your occupation：

⬜Government employees ⬜Sole trader

⬜Enterprise employee ⬜Student ⬜Retired ⬜Other

## 5.Your monthly income：

⬜5,000 yuan or less ⬜5,001– 10,000 yuan ⬜10,001– 20,000 yuan

⬜20,001– 30,000 yuan ⬜30,001 yuan or more

## 6.Have you visited the Mogao Grottoes before：

⬜Yes ⬜No

**Part 2: Measurement items for each variable**

Please answer the following questions according to your actual situation, and mark “√”(single choice) on the most suitable option in your opinion:

| **Items** | **Low←→High** | | | | | | |
| --- | --- | --- | --- | --- | --- | --- | --- |
| **Historical storytelling** | | | | | | | |
| The story of the place is engaging | 1 | 2 | 3 | 4 | 5 | 6 | 7 |
| The story of the place is very memorable | 1 | 2 | 3 | 4 | 5 | 6 | 7 |
| The story helps me to understand the history of the place | 1 | 2 | 3 | 4 | 5 | 6 | 7 |
| The story helps me to understand the people in the past | 1 | 2 | 3 | 4 | 5 | 6 | 7 |
| **Destination image** | | | | | | | |
| This place offers suitable accommodation | 1 | 2 | 3 | 4 | 5 | 6 | 7 |
| This place has high-quality infrastructure | 1 | 2 | 3 | 4 | 5 | 6 | 7 |
| The place offers attractive local food | 1 | 2 | 3 | 4 | 5 | 6 | 7 |
| This place has a standard of hygiene and cleanliness | 1 | 2 | 3 | 4 | 5 | 6 | 7 |
| The inhabitants of this place are interesting and friendly | 1 | 2 | 3 | 4 | 5 | 6 | 7 |
| This place has beautiful scenery | 1 | 2 | 3 | 4 | 5 | 6 | 7 |
| **Perceived value** | | | | | | | |
| Overall, I think the value of my travel experience is high | 1 | 2 | 3 | 4 | 5 | 6 | 7 |
| Compared to the time and energy I paid, I think I have received good value | 1 | 2 | 3 | 4 | 5 | 6 | 7 |
| Compared to the price I paid, I think I have received good value | 1 | 2 | 3 | 4 | 5 | 6 | 7 |
| **Revisit intention** | | | | | | | |
| I plan to visit the Mogao Grottoes again in the future | 1 | 2 | 3 | 4 | 5 | 6 | 7 |
| I will probably travel to the Mogao Grottoes again in the next five years | 1 | 2 | 3 | 4 | 5 | 6 | 7 |
| Given the opportunity,I will come to the Mogao Grottoes next time | 1 | 2 | 3 | 4 | 5 | 6 | 7 |
| **Place attachment** | | | | | | | |
| The Mogao Grottoes provided me with a unique tourism experience. | 1 | 2 | 3 | 4 | 5 | 6 | 7 |
| Compared to other tourist destinations, the tourism facilities and offerings at the Mogao Caves better cater to my needs. | 1 | 2 | 3 | 4 | 5 | 6 | 7 |
| Compared to other tourist destinations, I prefer Mogao Grottoes. | 1 | 2 | 3 | 4 | 5 | 6 | 7 |
| There is a strong sense of identification with the Mogao Grottoes. | 1 | 2 | 3 | 4 | 5 | 6 | 7 |
| I have a deep affection for the Mogao Caves. | 1 | 2 | 3 | 4 | 5 | 6 | 7 |
| Visiting the Mogao Caves is very meaningful to me. | 1 | 2 | 3 | 4 | 5 | 6 | 7 |
