# Supplementary material for: Exploring the influence of historical storytelling on cultural heritage tourists’ revisit intention: A case study of the Mogao Grottoes in Dunhuang
Source: PLoS One. 2024 Sep 19;19(9):e0307869. doi: 10.1371/journal.pone.0307869 (PMC11412526; doi:10.1371/journal.pone.0307869)
Supplement: Attachment — Submitted filename: Response to Reviewers.docx [file pone.0307869.s003.docx]

**Modification instructions**

Dear editors and reviewers of PLOS ONE:

Thank you for giving us the opportunity to submit a revised draft of the manuscript ‘Exploring the influence of historical storytelling on cultural heritage tourists' revisit intention: A case study of the Mogao Grottoes in Dunhuang’ for publication in the Journal of PLOS ONE. We appreciate the time and effort that you and the reviewers dedicated to providing feedback on our manuscript and are grateful for the insightful comments on and valuable improvements to our paper. We have incorporated most of the suggestions made by the reviewers. Those changes are highlighted in the manuscript. Please see below, in blue, for a point-by-point response to the reviewers’ comments and concerns. All page numbers refer to the revised manuscript file with tracked changes.

**Reviewer #1:**

1. **Lack of Theoretical Grounding: The study claims to be based on the SOR (Stimulus-Organism-Response) theory, but the application of this theory is not well-justified or thoroughly integrated throughout the manuscript. The connection between the theoretical framework and the hypotheses development is not clearly articulated, raising concerns about the study's theoretical underpinnings.**

**Revision note**: Thank you for the comments provided by the reviewer. In response to these suggestions, this study first reintegrated the literature review and hypothesis development sections. The specific application of the SOR model in this study is then explained in detail in the first part of the literature review. Finally, the application of the SOR model in this study is summarized in the hypothesis development section. The modified content is as follows (For details, please refer to manuscript pages 6-7 and 22).

In cultural heritage tourism research, scholars often focus on tourists' behavioral intentions and discuss their influencing factors ^[14]^. However, historical storytelling as a factor influencing tourist behavioral intentions, has received less attention. Therefore, it is necessary to further study the impact of historical storytelling on the tourism experience and revisit intentions of tourists at cultural heritage sites. Based on this, applying the SOR model to this study, for cultural heritage tourism destination visitors, historical storytelling is established as "stimulus" and revisit intention is established as "response". Additionally, some scholars have found that destination image and perceived value can serve as mediating mechanisms behind the behavioral intentions of tourists at cultural heritage sites ^[15-16]^. Therefore, this mediating mechanism influencing the behavioral intentions of tourists at cultural heritage sites can be used (as an organism) to further validate the applicability of the SOR framework. Furthermore, some scholars have introduced place attachment as a moderating variable to explore its moderating effect on tourist behavioral intentions, and have found that place attachment has a significant moderating effect between tourist satisfaction and revisit intentions ^[17]^. In summary, this study, based on the SOR theoretical model, considers historical storytelling as the "stimulus", destination image and tourist perceived value as the "organism", and tourist revisit intentions as the "response" to analyze the impact of historical storytelling on the revisit intentions of tourists at cultural heritage sites. (pages 6-7)

In conclusion, based on the above analysis and reasoning, this study has constructed a research model from the perspective of the SOR framework, which includes historical storytelling, destination image, perceived value, place attachment, and revisit intention (Fig 1). Furthermore, in this research model, destination image and perceived value play a mediating role, while place attachment has a moderating effect. (pages 22)

2. **Questionable Methodology: The data collection methods, including the online and offline survey distribution, are not described in sufficient detail. The sample size and composition are not adequately justified, and the potential biases or limitations of the sampling approach are not addressed. Additionally, the reliability and validity of the measurement scales used in the study are not thoroughly examined, which raises concerns about the robustness of the findings.**

**Revision note**: Thank you for the comments provided by the reviewers. In response to these suggestions, this study begins with a detailed description of the data collection methodology for this study in the Data Collection and Analysis section. Secondly, it provides proof of the adequacy of the sample size of this study. Then, this study clarified that the questionnaire data were obtained through convenience sampling. Finally, this study demonstrated Cronbach's alpha for each variable in the Results of reliability and convergent validity testing. The modifications made are as follows. (For further details, please refer to manuscript pages 25-26 and 29-30)

This study employs a convenience sampling method to conduct a survey on visitors at the Mogao Grottoes scenic area. This non-probability sampling strategy is deemed cost-effective and suitable for an on-site tourist survey in behavioral science research ^[73]^. While the target population and survey locations were intentionally chosen, the sampling process was spontaneous, which will reduce research bias ^[74]^. The data collection for this questionnaire is mainly based on the self-completion method. This study obtained data from two channels, online and offline. Firstly, the researchers distributed survey questionnaires to local tourists randomly at the Mogao Grottoes in Dunhuang during the peak tourist season. Data collection was conducted during two time periods to ensure the diversity of the data, from October 1, 2023 to October 3, 2023, and from January 1, 2024 to January 3, 2024. A total of 244 questionnaires were distributed and all were collected. Secondly, from February 3, 2024 to March 3, 2024, researchers distributed online survey questionnaires through the Wenjuanxing platform and received a total of 107 completed questionnaires. After excluding 26 incomplete questionnaires or those with identical answers for five consecutive questions, a total of 325 valid surveys were obtained, representing an effective response rate of 92.6%. There is no consensus among scholars regarding the selection of sample size. Comrey (1978) suggests that the minimum sample size should be at least 200, while Nunaly (1978) suggests that the sample size should be at least ten times the number of variables ^[75-76]^. Therefore, the 325 samples in this study meet the requirements of the above scholars, ensuring the accuracy and reliability of the model. This study used statistical software SPSS 26.0 and AMOS 26.0 to analyze the data from a total of 325 questionnaires collected online and offline. (pages 25-26)

**Table 2. Results of reliability and convergent validity testing.**

| **Construct measures** | **Factor Loading** | **Cronbach's α** | **AVE** | **CR** |
| --- | --- | --- | --- | --- |
| **Historical storytelling** |  | 0.936 | 0.791 | 0.938 |
| The story of the place is engaging | 0.937 |  |  |  |
| The story of the place is very memorable | 0.908 |  |  |  |
| The story helps me to understand the history of the place | 0.913 |  |  |  |
| The story helps me to understand the people in the past | 0.798 |  |  |  |
| **Destination image** |  | 0.882 | 0.532 | 0.872 |
| This place offers suitable accommodation | 0.742 |  |  |  |
| This place has high-quality infrastructure | 0.757 |  |  |  |
| The place offers attractive local food | 0.740 |  |  |  |
| This place has a standard of hygiene and cleanliness | 0.716 |  |  |  |
| The inhabitants of this place are interesting and friendly | 0.779 |  |  |  |
| This place has beautiful scenery | 0.737 |  |  |  |
| **Perceived value** |  | 0.778 | 0.601 | 0.817 |
| Overall, I think the value of my travel experience is high | 0.896 |  |  |  |
| Compared to the time and energy I paid, I think I have received good value | 0.747 |  |  |  |
| Compared to the price I paid, I think I have received good value | 0.666 |  |  |  |
| **Revisit intention** |  | 0.851 | 0.709 | 0.878 |
| I plan to visit the Mogao Grottoes again in the future | 0.913 |  |  |  |
| I will probably travel to the Mogao Grottoes again in the next five years | 0.680 |  |  |  |
| Given the opportunity, I will come to the Mogao Grottoes next time | 0.908 |  |  |  |
| **Place attachment** |  | 0.874 | 0.536 | 0.874 |
| The Mogao Grottoes provided me with a unique tourism experience. | 0.744 |  |  |  |
| Compared to other tourist destinations, the tourism facilities and offerings at the Mogao Caves better cater to my needs. | 0.686 |  |  |  |
| Compared to other tourist destinations, I prefer Mogao Grottoes. | 0.732 |  |  |  |
| There is a strong sense of identification with the Mogao Grottoes. | 0.757 |  |  |  |
| I have a deep affection for the Mogao Caves. | 0.723 |  |  |  |
| Visiting the Mogao Caves is very meaningful to me. | 0.750 |  |  |  |

**Note: CR = composite reliability; AVE = average variance extracted.**

3. **Narrow Scope and Limited Generalizability: The study focuses solely on the Mogao Grottoes in Dunhuang, which limits the generalizability of the findings to other cultural heritage tourism sites. The authors do not provide a clear rationale for selecting this specific case study, nor do they discuss the potential contextual factors that may have influenced the results.**

**Revision note**: Thank you for the comments provided by the reviewer. In response to these suggestions, first, the study explains the rationale for choosing the Mogao Caves in Dunhuang as the case site for this study in the Scope and Objects section. The long history and culture of Mogao Grottoes can provide a basis and convenience for us to study the influence of historical storytelling on tourists' revisit intention. Secondly, this study elaborates in the Limitations and Future Research Directions section that due to Dunhuang Mogao Grottoes' long history and innovative tourism experience programs, it may limit the generalizability of its findings. Whether this relationship holds true for other cultural heritage sites could be further explored in the future. The modified content is as follows. (Please refer to pages 23-24 and 46 of the paper for more details.)

This study takes the Mogao Grottoes in Dunhuang, a UNESCO World Heritage site, as a case study. Popularly known as the Thousand Buddha Caves, the Mogao Grottoes are hailed as the "most valuable cultural discovery of the 20th century" and the "Eastern Louvre". They are the largest and most abundant Buddhist art site in the world, possessing significant historical and cultural value as well as tourism value. Meanwhile, the Dunhuang Mogao Grottoes serve as a microcosm of the evolution of Chinese cave art, representing the longest-lasting, most rich in content, artistically exquisite, well-preserved, and most influential grotto group in China ^[66]^. In 1987, the Mogao Grottoes were designated as one of the first World Cultural Heritage sites in China, enjoying high international recognition and attracting a large number of visitors for sightseeing. This can provide a representative sample for empirical research on the tourist experience and revisit intentions of cultural heritage sites.

In Dunhuang, where the tourism industry accounts for a significant proportion of the tertiary sector at 63.33%, the Mogao Grottoes undoubtedly serve as the cornerstone of its tourism brand. The development of Mogao Grottoes tourism directly determines the prosperity or decline of Dunhuang's tourism economy. According to statistics from the cultural and tourism department of Dunhuang City, in 2023, the Dunhuang tourism industry, centered around the Mogao Grottoes, received a total of 16.82 million visitors and achieved tourism revenue of 15.6 billion yuan, both reaching historical highs ^[67]^. In recent years, the Dunhuang Research Institute has further developed cultural experiential projects to enhance the Dunhuang tourism experience and attract more visitors. For example, the digital Dunhuang project "Seeking Dunhuang" combines advanced game technologies such as 3D modeling and VR virtual reality scenes to lead visitors to "travel through" historical time and "explore" the thousand-year-old Dunhuang. Compared with other cultural heritage sites, the Mogao Grottoes have experienced thousands of years of history, where various cultures from ancient and modern times, both domestic and foreign, converge and blend. This long and ancient history provides an important cultural backdrop and ample historical materials for historical storytelling, as well as facilitates our study of the impact of historical storytelling on tourists' travel experiences and revisit intentions. (pages 23-24)

Finally, in terms of the research subject. This study takes the famous World Cultural Heritage site, the Mogao Grottoes in Dunhuang, as a case study for empirical research. Its long history and innovative tourism experience projects are likely to further enhance the effectiveness of historical storytelling, which may limit the generalization of the research findings to other cultural heritage sites. In future research, further exploration can be conducted on the impact of historical storytelling on tourists' travel experiences and revisiting intentions at other cultural heritage sites, in order to explore potential variations in the effectiveness of these strategies. (pages 46)

4. **Lack of Depth in Literature Review: The literature review section is relatively brief and lacks a comprehensive analysis of the existing research on historical storytelling, destination image, perceived value, place attachment, and revisit intention. The review does not adequately situate the current study within the broader context of cultural heritage tourism research, limiting the study's contribution to the field.**

**Revision note**: Thank you for the reviewer's comments. In response to these suggestions, firstly, this study adds some information on the five components of historical storytelling, destination image, perceived value, place attachment, revisit intention and the relationship between them in the literature review section. Secondly, some scholars' studies on these five components and their relationships in the context of cultural heritage tourism were added to the literature review process. The main modifications made are as follows (for further details, please refer to pages 8-9, 10-11, 13, 15, 17 and 18-22 of the paper).For more information, please refer to the main body of the paper.

In recent years, scholarly interest in storytelling has proliferated across diverse disciplines, including politics, communication, cognitive science, anthropology, organizational behavior, and marketing, with a particular resonance in the domain of marketing ^[20]^. Currently, the tourism industry and academia seem to widely acknowledge that storytelling is an effective communication tool that can attract potential tourists and promote and differentiate destinations ^[21]^. Recent research endeavors have delved into various aspects of storytelling, encompassing theoretical frameworks, research methodologies, and the impact of storytelling on destination image and tourist experiences. Zhang and Ramyah (2024) conducted a comprehensive literature review on the role of storytelling in destination marketing, revealing that scholars predominantly employ Heider's Balance Theory and Narrative Transportation Theory in their investigations, with the latter gaining more traction in recent years ^[20]^. Furthermore, it is observed that a majority of researchers favor qualitative approaches in studying storytelling, significantly outnumbering quantitative studies.

Furthermore, the examination of storytelling's influence on tourism destination image and visitor experience has consistently piqued the interest of researchers. For instance, Jo, Cha and Kim(2022) collected data from 259 tourists who experienced historical storytelling during their travels in South Korea, examining the influence of tourism storytelling on the tourism destination brand value, lovemarks, and relationship strength ^[22]^. Gemar, Sánchez-Teba and Soler(2022) utilized survey-based methods and count models to assess tourists' perceptions of historical storytelling, destination image, and their intention to engage in cultural activities ^[23]^. Furthermore, scholars have employed qualitative methodologies, such as interviews and observations, to explore the experiences and interpretations of tourists engaged with cultural heritage storytelling ^[2]^.

It is worth noting that recently some scholars have begun to explore the application of digital storytelling in the tourism industry. For example, Paivetal. (2023) used a gaming experiment to find that digital storytelling can not only evoke people's perception of environmental degradation, stimulate ecological anxiety, sadness, and loneliness, but also connect these emotions with views on sustainable actions and future practices ^[24]^. (pages 8-9)

Some scholars have discussed the factors influencing destination image. Balogu and McCleary (1999) categorized the influencing factors of destination image into personal factors and stimulus factors ^[30]^. Building on this, Beelli and Martin (2004) expanded their research and summarized the influencing factors of destination image as individual factors and information sources, with personal factors typically including demographic variables, travel motivations, and destination familiarity; while information source factors are generally divided into first-hand information sources (experience and sensation) and second-hand information sources (social media and official websites, etc.) ^[31]^. Of note, Wang et al. (2024) utilized innovation system theory and qualitative methods to explore the influencing factors and formation process of Cultural Inheritance-Based Innovation (CIBI) in Heritage Tourism Destinations (HTD), finding that CIBI is influenced by multilevel factors of the environment, government, enterprises, and public, encompassing basic innovation management conditions and sociocultural constraints ^[4]^.

Many scholars have also studied the impact of destination image on tourists' behavioral intentions, dividing it into three stages: pre-travel, during travel, and post-travel. We find that most scholars primarily focus on the influence of destination image on tourists' post-travel behavioral intentions ^[15]^. Gavurova et al. (2023) investigate the impact of tourism destination image on travelers' behavioral intentions from a transportation perspective, utilizing cluster analysis and regression analysis to quantify the relationship between road transport development indicators and visitor spending, revealing that traffic conditions significantly influence visitors' willingness to pay ^[32]^. Yang et al. (2024) explored the heterogeneous effects of different tourism advertising styles on cultural heritage sites. The study found that compared to direct tourism advertising, metaphorical tourism advertising can shape a more attractive image of the tourist destination, thus stimulating stronger visitation intentions among cultural heritage site visitors ^[14]^. (pages 10-11)

Tourist perceived value is the subjective evaluation made by tourists regarding the services and products obtained during the travel process, which can exert a positive effect on aspects such as tourist satisfaction and behavioral intentions ^[39-40]^. Furthermore, it also influences both the pre-purchase and post-purchase stages. Empirical research conducted by many scholars has demonstrated that high levels of tourist perceived value can positively affect their revisit intention. For example, Peng et al. (2023) explored the impact mechanism of tourists' perceived happiness on the revisit intention to traditional Chinese medicine cultural tourism destinations based on attachment theory ^[2]^. Through empirical research, it was found that when tourists perceive a higher level of happiness at cultural heritage sites, their revisit intention is significantly enhanced. Yao et al. (2020) took the case of Meizhou Island, the birthplace of China's first world-class intangible cultural heritage - Mazu belief, to explore the significant positive impact of tourist perceived value on revisit intention, with place attachment playing a partial mediating role ^[40]^. (pages 13)

In general, scholars have extensively studied the factors influencing tourists' revisit intention, and it has been found that the revisit intention is the result of a cumulative effect based on multiple factors, necessitating consideration of various influences. The influence of external characteristics tends to be secondary in comparison to the role of emotional factors. Therefore, it is necessary to prioritize the perceptions and tourism experiences of visitors, conducting in-depth analyses of their practical needs and psychological motivations, in order to effectively enhance their revisit intention. For example, Rasoolimanesh et al. (2021) used the case study of the world heritage city of Kashan in Iran to empirically investigate how visitor participation, authenticity, and destination image directly or indirectly affect heritage site visitors' revisit intention and electronic word-of-mouth (eWOM) intention through memorable tourism experiences (MTE) ^[50]^. (pages 15)

In addition, many tourism scholars have found that place attachment has a direct or indirect positive relationship with tourists' satisfaction, loyalty, and behavioral intentions. This study focuses on the impact of place attachment on tourists' revisit intention. Place attachment refers to the special emotions that people have towards a place due to various factors, and this special emotion is an important influencing factor in forming tourists' revisit intention, playing a facilitating role in their decision-making process ^[54]^. At the same time, cultural heritage sites are more likely to evoke special emotions in tourists due to their unique historical, cultural, and emotional values, thereby fostering a sense of place attachment and stimulating revisit intentions ^[2,40]^. (pages 17)

The image of a tourism destination reflects tourists' overall perception and impression of the destination, and a good destination image can effectively stimulate tourists' willingness to visit and influence their travel decisions ^[15]^. Zhang and Hou (2014) conducted an empirical study using the case of Hongcun, a World Cultural Heritage site in Anhui province, which elucidated that tourists' positive perceptions of a destination's image significantly encourage revisit intention ^[59]^. Furthermore, Lai et al. (2020) used Australia as an example to predict the behavioral intentions of potential Chinese tourists by combining cognitive and affective components of food imagery. The results showed that cognitive food imagery was more predictive of tourist behavioral intentions than affective imagery ^[60]^. Therefore, this study focuses primarily on the impact of destination cognitive imagery on tourists' travel experiences and behavioral intentions.

In addition, many scholars have recently studied the relationship between destination image and tourist perceived value. Through empirical research, they have found that destination image has a significant impact on perceived value, with destinations having a favorable image often exhibiting higher perceived value ^[61]^. Gu and Cui (2023) used Hangzhou, which possesses three world cultural heritage sites, as a case study and found that tourists' cognition of world cultural heritage significantly influences their perceived quality and perceived value ^[62]^. Therefore, this study proposes the following hypothesis: (pages 18-19)

Based on the aforementioned literature review, we discern a positive influence of tourists' perceived value on their revisit intentions, an effect that persists within the context of cultural heritage tourism ^[2]^. For example, Han (2015) takes the Ling Shan Buddhist cultural leisure resort in Wuxi as an example, and through empirical research, it is found that perceived value is the antecedent variable of satisfaction and behavioral intention, and the perceived value of cultural tourist destinations will directly affect their behavioral willingness ^[63]^. Therefore, this study proposes the following hypotheses: (pages 19-20)

Based on the previous assumptions, this study attempts to delve into the potential chain-mediated effects in the research model. Existing literature has analyzed the relationship between destination image, perceived value, and tourists' revisit intention, revealing that perceived value plays a mediating role between destination image and tourists' revisit intention ^[64]^. Meanwhile, many scholars tend to focus only on the overall perceived value when exploring the mediating role of perceived value between destination image and behavioral intention ^[16,40]^. Therefore, this study mainly focuses on the role of overall perceived value in the model.

Xu and Li (2018) studied the mechanism of the effect of destination image in Xinjiang, China on tourists' behavioral intentions, finding that perceived value plays a crucial role in the impact mechanism of destination image perception on tourists' behavioral intentions ^[16]^. However, this relationship has not yet been fully explored in the specific context of cultural heritage tourism. This study argues that historical storytelling can effectively enhance tourists' perception and views of a destination, thereby enabling tourists to experience higher value and ultimately strengthening their revisit intention. Therefore, this study considers historical storytelling as the antecedent variable, destination image and perceived value as the mediating variables, and revisit intention as the outcome variable, to delve into the potential chain-mediated effects. Thus, this study proposes the following hypotheses: (pages 20-21)

Furthermore, based on the literature review above, we can find that place attachment has a significant impact on the revisit intention ^[63]^. It is worth noting that Yun and Liu (2023) introduced place attachment as a moderating factor into the research framework, and found that place attachment can moderate the impact of perceived distance on tourists' behavioral intentions ^[65]^. Furthermore, they discovered that perceived value mediates the moderating effect of place attachment on tourists.

Place attachment is the connection formed between individuals and places through interaction, which can influence tourists' emotions and behaviors towards tourism destinations ^[52]^. In cultural heritage tourism, this attachment may become more profound and intricate due to the unique character and historical significance of the cultural heritage sites ^[2]^. Therefore, this study believes that different levels of place attachment will moderate tourists' revisit intention. This study suggests that when tourists have a high level of place attachment to the destination, historical storytelling will make it easier for them to develop a desire to revisit, and the mediating effect of destination image and perceived value between historical storytelling and revisit intention will also be strengthened. Therefore, this study proposes the following hypothesis: (pages 21-22)

5. **Insufficient Exploration of Mediating and Moderating Mechanisms: While the study examines the mediating and moderating effects, the explanations for these relationships are not well-developed. The discussion of the underlying mechanisms and the theoretical justifications for the proposed relationships are not sufficiently addressed, reducing the study's conceptual depth and the overall understanding of the complex interplay between the variables.**

**Revision note**: Thank you for the comments provided by the reviewer. In response to these suggestions, firstly, this study explores in detail the mediating role of destination image and perceived value, as well as the moderating role of place attachment in the SOR model in the first part of the literature review, and integrates this mediating mechanism into the SOR model as an "organism". Secondly, in the last part of the literature review, the hypothesis development further explains the role of destination image, perceived value and place attachment in the model. The modifications made are as follows (please refer to pages 6-7 and 20-22 of the paper for further details).

In cultural heritage tourism research, scholars often focus on tourists' behavioral intentions and discuss their influencing factors ^[14]^. However, historical storytelling as a factor influencing tourist behavioral intentions, has received less attention. Therefore, it is necessary to further study the impact of historical storytelling on the tourism experience and revisit intentions of tourists at cultural heritage sites. Based on this, applying the SOR model to this study, for cultural heritage tourism destination visitors, historical storytelling is established as "stimulus" and revisit intention is established as "response". Additionally, some scholars have found that destination image and perceived value can serve as mediating mechanisms behind the behavioral intentions of tourists at cultural heritage sites ^[15-16]^. Therefore, this mediating mechanism influencing the behavioral intentions of tourists at cultural heritage sites can be used (as an organism) to further validate the applicability of the SOR framework. Furthermore, some scholars have introduced place attachment as a moderating variable to explore its moderating effect on tourist behavioral intentions, and have found that place attachment has a significant moderating effect between tourist satisfaction and revisit intentions ^[17]^. In summary, this study, based on the SOR theoretical model, considers historical storytelling as the "stimulus", destination image and tourist perceived value as the "organism", and tourist revisit intentions as the "response" to analyze the impact of historical storytelling on the revisit intentions of tourists at cultural heritage sites. (pages 6-7)

Based on the previous assumptions, this study attempts to delve into the potential chain-mediated effects in the research model. Existing literature has analyzed the relationship between destination image, perceived value, and tourists' revisit intention, revealing that perceived value plays a mediating role between destination image and tourists' revisit intention ^[64]^. Meanwhile, many scholars tend to focus only on the overall perceived value when exploring the mediating role of perceived value between destination image and behavioral intention ^[16,40]^. Therefore, this study mainly focuses on the role of overall perceived value in the model.

Xu and Li (2018) studied the mechanism of the effect of destination image in Xinjiang, China on tourists' behavioral intentions, finding that perceived value plays a crucial role in the impact mechanism of destination image perception on tourists' behavioral intentions ^[16]^. However, this relationship has not yet been fully explored in the specific context of cultural heritage tourism. This study argues that historical storytelling can effectively enhance tourists' perception and views of a destination, thereby enabling tourists to experience higher value and ultimately strengthening their revisit intention. Therefore, this study considers historical storytelling as the antecedent variable, destination image and perceived value as the mediating variables, and revisit intention as the outcome variable, to delve into the potential chain-mediated effects. Thus, this study proposes the following hypotheses:

H7: The destination image plays an intermediary role between historical storytelling and tourists' revisit intention.

H8: Tourist perceived value plays an intermediary role between historical storytelling and tourists' revisit intention.

H9: The destination image and tourist perceived value play a mediating role in the relationship between historical storytelling and tourists' revisit intention.

Furthermore, based on the literature review above, we can find that place attachment has a significant impact on the revisit intention ^[63]^. It is worth noting that Yun and Liu (2023) introduced place attachment as a moderating factor into the research framework, and found that place attachment can moderate the impact of perceived distance on tourists' behavioral intentions ^[65]^. Furthermore, they discovered that perceived value mediates the moderating effect of place attachment on tourists.

Place attachment is the connection formed between individuals and places through interaction, which can influence tourists' emotions and behaviors towards tourism destinations ^[52]^. In cultural heritage tourism, this attachment may become more profound and intricate due to the unique character and historical significance of the cultural heritage sites ^[2]^. Therefore, this study believes that different levels of place attachment will moderate tourists' revisit intention. This study suggests that when tourists have a high level of place attachment to the destination, historical storytelling will make it easier for them to develop a desire to revisit, and the mediating effect of destination image and perceived value between historical storytelling and revisit intention will also be strengthened. Therefore, this study proposes the following hypothesis:

H10: Place attachment plays a positive moderating role in the impact of historical storytelling on the revisit intention.

H11: Place attachment positively moderates the mediating role of destination image between historical storytelling and revisit intention in tourism.

H12: Place attachment positively moderates the mediating role of perceived value in historical storytelling and revisit intentions for tourists.

In conclusion, based on the above analysis and reasoning, this study has constructed a research model from the perspective of the SOR framework, which includes historical storytelling, destination image, perceived value, place attachment, and revisit intention (Fig 1). Furthermore, in this research model, destination image and perceived value play a mediating role, while place attachment has a moderating effect. (pages 20-22)

6. **Incomplete Discussion of Implications: The practical implications of the study's findings are not thoroughly explored. The authors provide some general suggestions for destination managers, but the discussion lacks specific, actionable recommendations. Additionally, the study fails to address the potential limitations and future research directions, which would have strengthened the overall contribution of the manuscript.**

**Revision note**: Thank you for the comments provided by the reviewer. In response to these suggestions, first, the study added specific, actionable recommendations in three areas in the management implications section. Second, this study draws on the work of Lai et al. (2020), who found that cognitive images are better predictors of tourists' behavioral intentions than affective images. Meanwhile, many scholars tend to focus only on overall perceived value when exploring the mediating role of perceived value between destination image and behavioral intention. (Yao et al., 2020; Xu and Li, 2018) Meanwhile, it is pointed out in the Limitations and Future Research Directions section that this study aims to explore the influence of historical storytelling on cultural heritage tourists' travel experiences and revisit intentions from a macro perspective. Therefore, the key variables that best reflect this influence were selected for analysis in this study. The modified content is as follows (For details, please refer to manuscript pages 40-46).

**Management implications**

The results of this study have certain management implications for the development of the Mogao Grottoes tourism industry. It can not only guide tourism practitioners and destination managers to enhance visitor experiences and increase perceived value through specific strategies, but also provide certain references and insights for other cultural heritage sites.

The practical significance of this study mainly includes three points. First, through the results of this study, we found that historical storytelling has a significant impact on enhancing tourists' revisit intention. Therefore, in the development of cultural heritage tourism, it is important to thoroughly collect and research various local historical stories, such as religious legends, mythological stories, biographies, fables, and court life. These stories should then be cleverly combined with tourist experiences through tour guides, digital technology, and virtual reality (VR)/augmented reality (AR) techniques or more innovative means. This will showcase the destination's rich historical significance and cultural heritage to tourists, allowing them to immerse themselves in the cultural experience, enhance their satisfaction, and strive for a broader development space in the tourism market. For the Mogao Grottoes scenic area, first of all, it is necessary for the local government and the scenic area management department to establish a professional team responsible for collecting, researching, and verifying various local stories and historical legends; secondly, not only should professional training be provided to tour guides so that they can vividly and accurately tell the local historical stories, but efforts should also be made to develop the Mogao Grottoes tourism app to provide story interpretation, AR experiences, and other functions; finally, through economic incentive measures and honor selection, local residents should be encouraged to participate in the inheritance and storytelling of historical stories, in order to enhance the authenticity and interactivity of the tourist experience.

Secondly, according to this study, we found that destination cognitive image and perceived value have a significant chain-mediated effect between historical storytelling and tourists' revisit intentions. Furthermore, destination cognitive image and perceived value themselves have a significant positive impact on tourists' revisit intentions. This finding emphasizes the importance of integrating historical storytelling and destination image building in destination management. Therefore, destination managers can develop tourism products and upgrade scenic areas based on tourists' consumption preferences and purchasing behavior, aiming to enhance tourists' destination cognitive image and perceived value, thus improving the quality of scenic area services and revenue, and extending the tourism life cycle of the Dunhuang Mogao Grottoes scenic area. Specifically, the Mogao Grottoes tourist site could conduct in-depth market research to comprehend the distinct needs and preferences of various visitor segments. Based on the research outcomes, it could devise a diversified range of tourism products. For instance, targeting young tourists, innovative projects integrating technology and interactive experiences could be developed, such as Augmented Reality (AR) tours and Virtual Reality (VR) experiences, offering them an immersive and novel experience. For art and culture enthusiasts, tour packages centered around Dunhuang murals could be introduced, combining art appreciation, cultural lectures, and hands-on workshops, enabling visitors to gain a more profound understanding of Dunhuang's art heritage. At the same time, it is important to strengthen the level of scenic area services and create a high-quality tourism service environment. By analyzing the strengths and weaknesses in the marketing and service management of the Mogao Grottoes tourism scenic area, and summarizing corresponding optimization strategies, the level of tourism scenic area service management can be improved, and its brand competitiveness can be enhanced. For example, the management department should increase funding and upgrade the infrastructure of the scenic area, such as improving restroom facilities, adding rest areas, and providing more information signs, in order to enhance the comfort of tourists during their visits. In addition, it is necessary to establish a standardized complaint handling process based on feedback from tourists, clearly defining the steps of complaint reception, recording, investigation, response, and feedback, to ensure transparency in the process and let tourists know how their complaints will be addressed.

Finally, the research results also emphasize the significant moderating role of place attachment between historical storytelling and tourists' revisit intention. This requires cultural heritage sites to pay attention to cultivating visitors' place attachment emotions in the management and development process, in order to promote visitors' tourism experience, satisfaction, and revisit intention, and thus promote the sustainable development of cultural heritage tourism. Managers should pay particular attention to showcasing the unique culture of Mogao Grottoes and its distinctive tourism products in tourists' travel experiences, allowing them to obtain a one-of-a-kind tourism experience and creating a competitive advantage through differentiation. As a representative of Dunhuang culture, Mogao Grottoes possesses abundant historical and cultural heritage resources. Integrating this unique cultural resource into tourists' travel experiences is conducive to fostering a sense of connection between people and the land, enhancing tourists' emotional attachment to the place, and thereby stimulating their revisit intention. First, the scenic area can design a series of cultural experience activities, such as Dunhuang mural copying, Silk Road history lectures, Dunhuang dance workshops, etc., to allow visitors to personally experience the unique charm of Dunhuang culture; secondly, managers can make full use of social media platforms to encourage visitors to share their experiences and stories at the Mogao Grottoes, and enhance visitors' attachment to the Mogao Grottoes through user-generated content; finally, the Mogao Grottoes can also provide personalized services for visitors, such as inviting them to attend the opening of new exhibitions and cultural lectures at the Mogao Grottoes, or offering special discounts to returning visitors, allowing visitors to feel the special attention of the Mogao Grottoes to them, effectively enhancing visitors' emotional attachment to the Mogao Grottoes and stimulating their revisit intention.

**Limitations and future research directions**

The findings of this study provide relevant insights and perspectives on the relationships between historical storytelling, destination image, perceived value, place attachment, and tourists' revisit intention. However, there are still some limitations.

Firstly, in terms of research methods. The questionnaire data obtained in this study mainly relies on self-administered surveys by tourists. Given that the responses to the survey may be influenced by participants' cultural background, regional of origin, educational background, mood, cognition, and experiences, the answers provided by the respondents are inherently constrained to some extent. In future research, it could be beneficial to consider incorporating more objective data collection methods to enhance the reliability of the study. For example, using structured interviews to have face-to-face communication with tourists, delving deeper into their perspectives and opinions; or employing observational methods to collect data by directly observing tourists' behaviors and phenomena; additionally, combining big data analysis by utilizing existing datasets and data mining techniques to gather information.

Secondly, in terms of research content. This study mainly focuses on the destination cognitive image and tourists' overall perceived value, without conducting in-depth analysis on other dimensions of destination image and perceived value. The aim is to explore the influence of historical storytelling on the tourist experience and revisit intention of cultural heritage site visitors from a macro perspective. In future research, further exploration can be conducted on the emotional image and overall image within the destination image, as well as other dimensions such as transaction value and acquisition value in perceived value, in order to reveal the influence of historical storytelling on destination image and tourist perceived value from a more micro perspective. Furthermore, this study examined the moderating effect of place attachment, and found that it does not have a significant moderating effect on the mediating role of destination cognitive image and perceived value. In future research, other related moderating variables such as tourism involvement, gender, emotions, and novelty-seeking motivation can be explored.

Finally, in terms of the research subject. This study takes the famous World Cultural Heritage site, the Mogao Grottoes in Dunhuang, as a case study for empirical research. Its long history and innovative tourism experience projects are likely to further enhance the effectiveness of historical storytelling, which may limit the generalization of the research findings to other cultural heritage sites. In future research, further exploration can be conducted on the impact of historical storytelling on tourists' travel experiences and revisiting intentions at other cultural heritage sites, in order to explore potential variations in the effectiveness of these strategies. (pages 40-46)

**Reviewer #2:**

1. **However, the paper could benefit from the inclusion of more citations to support its claims and provide a stronger theoretical foundation. Referencing existing literature on cultural heritage tourism, storytelling, and tourist behavior would enhance the paper's academic rigor and situate its findings within the broader research context.**

**Recommended article for citations:**

**https://journals.economic-research.pl/eq/article/view/2398/2086**

**and https://www.tandfonline.com/doi/full/10.1080/14616688.2023.2224043**

**and https://www.tandfonline.com/doi/full/10.1080/01490400.2021.1908193 Etc.**

**Revision note**: Thank you for the comments provided by the reviewer. I have read the above three references, which are very helpful for my research. In response to these suggestions, first, this study cites the three references mentioned above in the revised manuscript. Second, this study expands on the literature review, especially the studies related to historical storytelling, and incorporates them into the study of cultural heritage tourism. The modifications made are as follows (please refer to pages 7-9, 11 and 17-18 of the paper for further details).

Storytelling is a social phenomenon influenced by psychology, sociology, marketing, tourism, and behavioral sciences. Palombini (2017) defines storytelling as the narration of events that happen to certain individuals, leading to changes in the situation ^[18]^. Building upon this, historical storytelling can be simply understood as the telling of historical events related to surrounding objects in appropriate settings. As scholars delve deeper into the study of historical storytelling, some have started to apply them in tourism research, aiming to enhance tourists' understanding of destinations, improve their perceived value, and stimulate their interest in travel ^[5]^. Go and Govers (2012) found that storytelling greatly attracts tourists emotionally and cognitively, positively impacting their engagement and satisfaction ^[19]^. In cultural heritage tourism, historical storytelling involves telling stories, anecdotes, and interesting facts related to cultural heritage vividly and engagingly. It is worth noting that Palombini (2017) emphasizes that storytelling in cultural heritage focuses on imparting specific knowledge about the historical events, cultural values, and social background of the depicted cultural heritage ^[18]^. Against the backdrop of the flourishing development of cultural heritage tourism, tourists' perception of the cultural heritage determines their cognitive image and value judgment of the destination, subsequently influencing their behavioral intentions. Historical storytelling serves as a crucial means to enhance tourists' cultural perception.

In recent years, scholarly interest in storytelling has proliferated across diverse disciplines, including politics, communication, cognitive science, anthropology, organizational behavior, and marketing, with a particular resonance in the domain of marketing ^[20]^. Currently, the tourism industry and academia seem to widely acknowledge that storytelling is an effective communication tool that can attract potential tourists and promote and differentiate destinations ^[21]^. Recent research endeavors have delved into various aspects of storytelling, encompassing theoretical frameworks, research methodologies, and the impact of storytelling on destination image and tourist experiences. Zhang and Ramyah (2024) conducted a comprehensive literature review on the role of storytelling in destination marketing, revealing that scholars predominantly employ Heider's Balance Theory and Narrative Transportation Theory in their investigations, with the latter gaining more traction in recent years ^[20]^. Furthermore, it is observed that a majority of researchers favor qualitative approaches in studying storytelling, significantly outnumbering quantitative studies.

Furthermore, the examination of storytelling's influence on tourism destination image and visitor experience has consistently piqued the interest of researchers. For instance, Jo, Cha and Kim(2022) collected data from 259 tourists who experienced historical storytelling during their travels in South Korea, examining the influence of tourism storytelling on the tourism destination brand value, lovemarks, and relationship strength ^[22]^. Gemar, Sánchez-Teba and Soler(2022) utilized survey-based methods and count models to assess tourists' perceptions of historical storytelling, destination image, and their intention to engage in cultural activities ^[23]^. Furthermore, scholars have employed qualitative methodologies, such as interviews and observations, to explore the experiences and interpretations of tourists engaged with cultural heritage storytelling ^[2]^.

It is worth noting that recently some scholars have begun to explore the application of digital storytelling in the tourism industry. For example, Paiva et al. (2023) used a gaming experiment to find that digital storytelling can not only evoke people's perception of environmental degradation, stimulate ecological anxiety, sadness, and loneliness, but also connect these emotions with views on sustainable actions and future practices ^[24]^. (pages 7-9)

Many scholars have also studied the impact of destination image on tourists' behavioral intentions, dividing it into three stages: pre-travel, during travel, and post-travel. We find that most scholars primarily focus on the influence of destination image on tourists' post-travel behavioral intentions ^[15]^. Gavurova et al. (2023) investigate the impact of tourism destination image on travelers' behavioral intentions from a transportation perspective, utilizing cluster analysis and regression analysis to quantify the relationship between road transport development indicators and visitor spending, revealing that traffic conditions significantly influence visitors' willingness to pay ^[32]^. Yang et al. (2024) explored the heterogeneous effects of different tourism advertising styles on cultural heritage sites. The study found that compared to direct tourism advertising, metaphorical tourism advertising can shape a more attractive image of the tourist destination, thus stimulating stronger visitation intentions among cultural heritage site visitors ^[14]^. (pages 11)

Storytelling is ubiquitous in every aspect of life, as it can help people connect with each other and cultivate empathy, leading individuals to attempt to "feel the world in the same way as the characters in the story" ^[55-56]^. In the digital age, historical storytelling is considered an effective means to enhance the reputation of destinations ^[57]^. When tourism service personnel tell historical stories at cultural heritage sites, they create immersive and memorable experiences for tourists, connecting them with the local social and cultural context, providing higher quality tourism services, and helping tourists better understand the destination, stimulating their interest in tourism, and increasing their participation and satisfaction ^[6]^. Revisit intention usually refers to the likelihood of tourists returning to the destination, with factors influencing revisit intention mainly including satisfaction, destination image, perceived value, etc. ^[58]^. Although the presence of historical storytelling is not among the numerous influencing factors, existing research has found that historical storytelling has a positive impact on the tourist experience and perceived value of cultural heritage sites ^[6]^. Therefore, this study believes that telling historical stories at cultural heritage sites can provide visitors with a better tourism experience, enhance visitors' cognitive image, perceived value, and revisit intention. Thus, this study proposes the following hypotheses: (pages 17-18)

2. **Additionally, the English language and overall readability of the paper could be improved. Some sections contain grammatical errors and awkward phrasing that could be polished for clarity and professionalism. A thorough revision for language accuracy and coherence would greatly enhance the paper’s readability and impact. For instance, the sentence "Visitor perception experience has always been a focal point of academic inquiry" could be revised to "Visitor perception has long been a focal point of academic inquiry." Refining the abstract and other sections for smoother flow and clearer articulation of ideas would make the paper more accessible to a broader audience.**

**Revision note**: Thank you for the comments provided by the reviewer. In response to these suggestions, this study used specialized grammar proofreading software to revise and embellish the grammar of the sentences. In addition, this study made further changes and refinements to the sentences in the abstract and other parts of the study. The specific modifications to the abstract are as follows (please refer to pages 1-2 of the paper for further details). For more information, please refer to the main body of the paper.

The revisit intention of tourists has long been a focal point of academic inquiry. However, there is still insufficient research on the antecedents of revisit intention from the perspectives of historical storytelling, destination image and perceived value. Taking the Mogao Grottoes in Dunhuang, a UNESCO World Heritage Site, as a case study, this paper, based on stimulus–organism–response (SOR) theory, examines the impact of historical storytelling on the destination image, perceived value, and revisit intention. Additionally, it further explores the mediating role of destination image and perceived value, as well as the moderating effect of place attachment in this chain. The research findings indicate that: (1) Historical storytelling significantly enhances tourists' perception of the tourism experience and revisit intention; (2) The study supports the mediating effect of destination image and perceived value; (3) Place attachment has a significant positive moderating effect between historical storytelling and revisit intention. Effective historical storytelling can significantly enhance destination image and perceived value, improve tourists' participation and satisfaction in tourism, stimulate revisit intention, and promote the sustainable development of tourist destinations. These findings enrich the research content of cultural heritage tourism, providing valuable suggestions for improving the management level of cultural heritage tourism attractions and increasing visitors' revisit intention. (pages 1-2)

3. **In conclusion, "Exploring the Influence of Historical Storytelling on Cultural Heritage Tourists' Revisit Intention" is a significant and valuable contribution to the field of cultural heritage tourism. By addressing the need for more citations and improving the English language quality, the paper can further elevate its impact and utility for researchers, tourism managers, and policymakers. The innovative focus on historical storytelling as a tool for enhancing tourist engagement and promoting sustainable tourism development is particularly noteworthy, providing a solid foundation for future research and practical applications in the tourism industry.**

**Revision note**: I greatly appreciate your constructive comments during the review process, which not only help to improve my current research, but also provide important guidance for my future academic exploration. Thank you again for your professional review and valuable time.

**Reviewer #3:**

1. **The minimum number was provided in the sample selection of the research, but since it was a multivariate research, a sample over 384 would have been more appropriate. It is recommended that sampling adequacy be explained by power analysis or another method.**

**Revision note**: Thank you for the comments provided by the reviewer. In response to these suggestions, this study provides evidence of the adequacy of the sample size of this study in the Data Collection and Analysis section. The modifications made are as follows (please refer to pages 25-26 of the paper for further details).

This study employs a convenience sampling method to conduct a survey on visitors at the Mogao Grottoes scenic area. This non-probability sampling strategy is deemed cost-effective and suitable for an on-site tourist survey in behavioral science research ^[73]^. While the target population and survey locations were intentionally chosen, the sampling process was spontaneous, which will reduce research bias ^[74]^. The data collection for this questionnaire is mainly based on the self-completion method. This study obtained data from two channels, online and offline. Firstly, the researchers distributed survey questionnaires to local tourists randomly at the Mogao Grottoes in Dunhuang during the peak tourist season. Data collection was conducted during two time periods to ensure the diversity of the data, from October 1, 2023 to October 3, 2023, and from January 1, 2024 to January 3, 2024. A total of 244 questionnaires were distributed and all were collected. Secondly, from February 3, 2024 to March 3, 2024, researchers distributed online survey questionnaires through the Wenjuanxing platform and received a total of 107 completed questionnaires. After excluding 26 incomplete questionnaires or those with identical answers for five consecutive questions, a total of 325 valid surveys were obtained, representing an effective response rate of 92.6%. There is no consensus among scholars regarding the selection of sample size. Comrey (1978) suggests that the minimum sample size should be at least 200, while Nunaly (1978) suggests that the sample size should be at least ten times the number of variables ^[75-76]^. Therefore, the 325 samples in this study meet the requirements of the above scholars, ensuring the accuracy and reliability of the model. This study used statistical software SPSS 26.0 and AMOS 26.0 to analyze the data from a total of 325 questionnaires collected online and offline. (pages 25-26)

2. **The research findings are quite meaningful. The results are supported by the literature, but the striking results can be emphasized a little more. For example, since historical storytelling is an important concept, it should be supported with concrete storytelling.**

**Revision note**: Thank you for the comments provided by the reviewer. In response to these suggestions, this study further explains what concrete storytelling can be supported by historical storytelling in the Management Implications section. For example, religious legends, mythological stories, biographies, fables, and court life. The modified content is as follows (For details, please refer to manuscript pages 40-41).

The practical significance of this study mainly includes three points. First, through the results of this study, we found that historical storytelling has a significant impact on enhancing tourists' revisit intention. Therefore, in the development of cultural heritage tourism, it is important to thoroughly collect and research various local historical stories, such as religious legends, mythological stories, biographies, fables, and court life. These stories should then be cleverly combined with tourist experiences through tour guides, digital technology, and virtual reality (VR)/augmented reality (AR) techniques or more innovative means. This will showcase the destination's rich historical significance and cultural heritage to tourists, allowing them to immerse themselves in the cultural experience, enhance their satisfaction, and strive for a broader development space in the tourism market. For the Mogao Grottoes scenic area, first of all, it is necessary for the local government and the scenic area management department to establish a professional team responsible for collecting, researching, and verifying various local stories and historical legends; secondly, not only should professional training be provided to tour guides so that they can vividly and accurately tell the local historical stories, but efforts should also be made to develop the Mogao Grottoes tourism app to provide story interpretation, AR experiences, and other functions; finally, through economic incentive measures and honor selection, local residents should be encouraged to participate in the inheritance and storytelling of historical stories, in order to enhance the authenticity and interactivity of the tourist experience. (pages 40-41)

3. **Particular emphasis should be placed on the regulatory role of place attachment, with literature support.**

**Revision note**: Thank you for the comments provided by the reviewer. In response to these suggestions, this study further elaborates on the positive moderating effect of place attachment on the revisiting intentions of tourists at cultural heritage sites in the conclusion and discussion section, and provides relevant literature support. The modified content is as follows (For details, please refer to manuscript pages 38).

Finally, according to the established hypothesis H10, place attachment plays a significant positive moderating role between historical storytelling and revisit intention. This study was conducted in the context of cultural heritage tourism, revealing that the impact of historical storytelling on revisit intention varies across different levels of place attachment. When tourists have a higher level of place attachment, the influence of historical storytelling on their revisit intention is strengthened. The non-confirmation of Hypotheses H11 and H12 suggests that place attachment does not exert a significant moderating influence on the mediating effect of destination cognitive image and perceived value in the relationship between historical storytelling and revisit intention. This indicates that in cultural heritage tourism, place attachment is an important factor influencing tourists' revisit intention, but its role is mainly reflected in directly enhancing the attractiveness of historical storytelling to tourists, rather than indirectly affecting tourists' revisit intention by adjusting other variables (such as destination cognitive image and perceived value). This is also similar to the research results of Hu, Wang, and Yun et al., suggesting that place attachment may have a certain moderating and promoting effect on revisit intention ^[17,65,83]^. Wang et al. (2022) found that by introducing place attachment as a moderating variable, place attachment plays a positive moderating role in the relationship between pro-environmental behavior and revisit intention ^[83]^. (pages 38)

**Reviewer #4:**

1. **The author needs to revise the abstract, and the first part briefly writes down the background and purpose.**

**Revision note**: Thank you for the comments provided by the reviewers. In response to these suggestions, the abstract of this study has been revised and the background and purpose of the study are briefly described in the first section. The modifications made are as follows. (For further details, please refer to manuscript pages 1-2)

**Abstract**

The revisit intention of tourists has long been a focal point of academic inquiry. However, there is still insufficient research on the antecedents of revisit intention from the perspectives of historical storytelling, destination image and perceived value. Taking the Mogao Grottoes in Dunhuang, a UNESCO World Heritage Site, as a case study, this paper, based on stimulus–organism–response (SOR) theory, examines the impact of historical storytelling on the destination image, perceived value, and revisit intention. Additionally, it further explores the mediating role of destination image and perceived value, as well as the moderating effect of place attachment in this chain. The research findings indicate that: (1) Historical storytelling significantly enhances tourists' perception of the tourism experience and revisit intention; (2) The study supports the mediating effect of destination image and perceived value; (3) Place attachment has a significant positive moderating effect between historical storytelling and revisit intention. Effective historical storytelling can significantly enhance destination image and perceived value, improve tourists' participation and satisfaction in tourism, stimulate revisit intention, and promote the sustainable development of tourist destinations. These findings enrich the research content of cultural heritage tourism, providing valuable suggestions for improving the management level of cultural heritage tourism attractions and increasing visitors' revisit intention. (pages 1-2)

2. **Add a reference to the first paragraph of the introduction regarding the number of cultural heritages worldwide. Where does the first sentence of the second paragraph of the introduction come from?**

**Revision note**: Thank you for the comments provided by the reviewer. In response to these suggestions, this study adds references to the number of cultural heritages worldwide and to the first sentence of the second paragraph of the introduction. The modified content is as follows (For details, please refer to manuscript pages 2-3).

As per the resolution of the 45th session of the World Heritage Committee, by October 2023, the total number of global heritage sites expanded to 1199, comprising 993 cultural heritage sites ^[1]^. (pages 2)

Historical storytelling, as a propaganda medium in the tourism process, can have a significant impact on tourists' participation and experience ^[5-6]^. (pages 3)

3. **Please note the full name of the acronym SOR theory after its first appearance. It is suggested that the third and fourth paragraphs of the introduction be merged, followed by a condensed version of the introduction to make this part as concise as possible.**

**Revision note**: Thank you for the comments provided by the reviewers. In response to these suggestions, this study added the full name of the SOR theory to the abstract section. Second, this study merged the third and fourth paragraphs of the introduction and streamlined the introduction as a whole. The introduction was modified as follows. (For further details, please refer to manuscript pages 2-4)

**Introduction**

Cultural heritage tourism is an integral component of heritage tourism. As per the resolution of the 45th session of the World Heritage Committee, by October 2023, the total number of global heritage sites expanded to 1199, comprising 993 cultural heritage sites ^[1]^. With the increasing internal demand for high-quality development of cultural tourism and the intensifying competition among similar tourist destinations, cultural heritage tourism destinations are also facing many challenges, especially in terms of how to maintain customer numbers and competitiveness ^[2]^. Han & Hyun (2015) found that attracting repeat visitors can make destinations more cost-effective and beneficial for the sustainable development of cultural heritage sites ^[3]^. However, current research on cultural heritage tourism mainly focuses on the protection and development of cultural heritage itself, and there is not enough comprehensive research on tourists' subjective behavioral intentions from the perspective of cultural heritage site visitors ^[4]^. Therefore, it is necessary for us to study how to enhance the tourist perception and revisit intention of cultural heritage sites.

Historical storytelling, as a propaganda medium in the tourism process, can have a significant impact on tourists' participation and experience ^[5-6]^. Telling historical stories is considered a powerful tool to make historical events more attractive, while also effectively attracting tourists and enhancing their competitive advantage ^[7-8]^. Cultural heritage sites often have a long history and unique cultural heritage, providing important cultural scenes and ample historical materials for historical storytelling. Appropriate historical storytelling can help visitors better understand the development process of cultural heritage sites and the local distinctive culture, thus establishing an emotional connection between tourists and destinations, which is crucial for enhancing tourists' travel experience. Current research has applied historical storytelling to the tourism industry, exploring its impact on tourist emotional engagement and destination branding, and has found that it can indeed effectively enhance tourist perceptual experiences in different tourism settings ^[8-9]^. Leong, Yeh, Zhou, Hung, and Huan(2024) examined the impact of historical storytelling on tourists' perception of education, entertainment, experience, and emotional value in cultural heritage tourism sites through tour guide interaction and authentic place as intermediaries ^[6]^. However, they failed to link these variables to travel outcomes, such as revisit intentions and word-of-mouth intentions. It is worth noting that the impact mechanism of historical storytelling on tourists' revisit intention has not been fully explored, requiring further theoretical exploration and empirical research.

Based on the SOR theory, this study aims to explore the influence of historical storytelling on tourists' revisit intentions in the context of cultural heritage tourism, in order to improve the quality of tourism services in scenic areas and enhance tourists' travel experiences, increase tourists' sense of participation and satisfaction, and stimulate tourists' revisit intentions. This study makes a significant contribution to the field of cultural heritage tourism. Firstly, starting from historical storytelling, a research model on the influence of historical storytelling on tourists' revisit intention was constructed, expanding the relevant research on tourists' revisit intention, enriching the research content of cultural heritage tourism, and providing alternative perspectives for the study of cultural heritage tourism. Secondly, this study examines the mediating effect of destination image and perceived value in the model, as well as the moderating effect of place attachment. This not only helps to theoretically understand how historical storytelling influences tourists' revisit intention, but also assists tourism destination managers in formulating appropriate development strategies, providing valuable insights for scholars and tourism practitioners. (pages 2-4)

4. **It is recommended to compress the content of the literature review in Chapter II. The literature review should be closely centered on the topic of the paper. The relationship between the literature review of several sections is briefly introduced in the first paragraph of chapter 2.**

**Revision note**: Thank you for the comments provided by the reviewer. In response to these suggestions, this study reorganized the content of the literature review by focusing on the topic of the paper. The relationships between the five variables in this study's model are presented in the first part of the literature review, the SOR model. The specific modifications to the elaboration of the relationship between the variables are as follows (please refer to pages 6-7 of the paper for further details). For more information, please refer to the main body of the paper.

In cultural heritage tourism research, scholars often focus on tourists' behavioral intentions and discuss their influencing factors ^[14]^. However, historical storytelling as a factor influencing tourist behavioral intentions, has received less attention. Therefore, it is necessary to further study the impact of historical storytelling on the tourism experience and revisit intentions of tourists at cultural heritage sites. Based on this, applying the SOR model to this study, for cultural heritage tourism destination visitors, historical storytelling is established as "stimulus" and revisit intention is established as "response". Additionally, some scholars have found that destination image and perceived value can serve as mediating mechanisms behind the behavioral intentions of tourists at cultural heritage sites ^[15-16]^. Therefore, this mediating mechanism influencing the behavioral intentions of tourists at cultural heritage sites can be used (as an organism) to further validate the applicability of the SOR framework. Furthermore, some scholars have introduced place attachment as a moderating variable to explore its moderating effect on tourist behavioral intentions, and have found that place attachment has a significant moderating effect between tourist satisfaction and revisit intentions ^[17]^. In summary, this study, based on the SOR theoretical model, considers historical storytelling as the "stimulus", destination image and tourist perceived value as the "organism", and tourist revisit intentions as the "response" to analyze the impact of historical storytelling on the revisit intentions of tourists at cultural heritage sites. (pages 6-7)

**In addition, the reference part of this article has been revised and supplemented. Nineteen relevant articles were added and thirteen original articles were deleted.**

**Additional literature includes:**

[1]Extended 45th session of the World Heritage Committee. Riyadh, Kingdom of Saudi Arabia, 2023 [cited 2014 Jun 11]. Available from: https://whc.unesco.org/archive/2023/whc23-45com-19-en.pdf.
[2]Zi-Ting Yang, Wen-Qi Ruan, Shu-Ning Zhang, Zi-Chin Cheng. Revealing the metaphorical code: Heterogeneous effects and mechanisms of tourism advertising style in cultural heritage sites. Journal of Hospitality and Tourism Management. 2024; 59: 263-275. DOI: 10.1016/j.jhtm.2024.05.001
[3]M. Choi, R. Law, C.Y. Heo. Shopping destinations and trust-tourist attitudes: Scale development and validation. Tourism Management. 2016; 54: 490-501. DOI: 10.1016/j.tourman.2016.01.005
[4]R.S. Nickerson. Confirmation bias: A ubiquitous phenomenon in many guises. Review of General Psychology. 1998; 2 (2): 175-220. DOI: 10.1037/1089-2680.2.2.175
[5]Comrey, A.L. Common methodological problems in factor analytic studies. Journal of Consulting and Clinical psychology. 1978; 46: 648-659. DOI: 10.1037/0022-006X.46.4.648
[6]Xi Zhang, T. Ramayah. Solving the mystery of storytelling in destination marketing: A systematic review. Journal of Hospitality and Tourism Management. 2024; 59: 222-237. DOI: 10.1016/j.jhtm.2024.04.013
[7]T. Chautard, I. Collin-Lachaud. Introducing the storytelling analysis methodology in marketing: Principles, contributions and implementation. Recherche et Applications en Marketing. 2019; 34 (3): 27-46. DOI: 10.1177/2051570719841225
[8]Gavurova, B., Rigelsky, M., Mikeska, M.. Relationships between road transport indicators and expenditure of visitors in the context of European countries' tourism competitiveness. Equilibrium. 2023; 18 (2): 393–418. DOI: 10.24136/eq.2023.012
[9]Paiva, D., Carvalho, L., Brito-Henriques, E., Sousa, A.M., Soares, A.L., Azambuja, S.T. Digital storytelling and hopeful last chance tourism experiences. Tourism Geographies. 2023; 25 (5): 1428–1444. DOI: 10.1080/14616688.2023.2224043
[10]Calvi, L., Hover, M. Storytelling for Mythmaking in Tourist Destinations. Leisure Sciences. 2021; 43 (6): 630–643. DOI: 10.1080/01490400.2021.1908193
[11]Seyhmus Baloglu, Mccleary, K.W. A model of destination image formation. Annals of Tourism Research. 1999; 26 (4): 868–897. DOI: 10.1016/S0160-7383(99)00030-4
[12]Beerli, A.; Martin, J.D. Factors influencing destination image. Annals of Tourism Research. 2004; 31 (3): 657–681. DOI: 10.1016/j.annals.2004.01.010
[13]Lai, M.Y.; Wang, Y.; Khoo–Lattimore, C. Do food image and food neophobia affect tourist intention to visit adestination? The case of Australia. Journal of Travel Research. 2020; 59 (5): 928–949. DOI: 10.1177/0047287519867144
[14]Kwun, J. W., Oh, H. Effects of Brand, Price, and Risk on Customers’ Value Perceptions and Behavioral Intentions in the Restaurant Industry. Journal of Hospitality & Leisure Marketing, 2004; 11 (1): 31–49. DOI: 10.1300/J150v11n01_03
[15]Jinshi Fan. The management and the monitoring system for the nature of the world cultural heritage site. Dunhuang Research. 2008; (6): 1-5. DOI: 10.3969/j.issn.1000-4106.2008.06.001
[16]S. Mostafa Rasoolimanesh，Siamak Seyfi，C.Michael Hall，Pezhman Hatamifar. Understanding memorable tourism experiences and behavioural intentions of heritage tourists. Journal of Destination Marketing &Management. 2021; 21: 100621. DOI: 10.1016/j.jdmm.2021.100621
[17]Mei-Yu Wang, Yong-Quan Li, Wen-Qi Ruan, Shu-Ning Zhang, Rui Li. Influencing factors and formation process of cultural inheritance-based innovation at heritage tourism destinations. Tourism Management. 2024; 100: 104799. DOI: 10.1016/j.tourman.2023.104799
[18]Yao, D.; Zhang, K.; Wang, L.; Law, R.; Zhang, M. From Religious Belief to Intangible Cultural Heritage Tourism: A Case Study of Mazu Belief. Sustainability. 2020; 12 (10): 4229. DOI:10.3390/su12104229
[19]Gu Yaqing, Cui Fengjun. The impact of World Cultural Heritage Sites' cognition on visitor satisfaction and loyalty:Taking Hangzhou as an example. World Regional Studies. 2023; 32(05): 138-147. DOI: 10.3969/j.issn.1004-9479.2023.05.20220054

**Deleted literature included:**

[1] Herzog, H. Behavioral science concepts for analyzing the consumer. Mark. Behav. Sci. 3(1963), pp. 76–86.
[2] Jie Li, Zhen Lu.On the image of tourism destinations and the strategy of tourism destination image.[J].Journal of Business Economics, 6(1996), pp. 62-65.
[3] Ryan, C., Cave, J. Structuring destination image: A qualitative approach[J].Journal of Travel Research, 44(2005), pp. 143-150.
[4] Wang, C.; Qu, H.; Hsu, M.K. Toward an integrated model of tourist expectation formation and gender difference. [J]. [Tourism Management](https://www.sciencedirect.com/journal/tourism-management), [54](https://www.sciencedirect.com/journal/tourism-management/vol/54/suppl/C)(2016), pp. 58-71.
[5] W.K.Tan, C.E.Wu. An investigation of the relationships among destination familiarity,destination image and future visit intention Journal of Destination Marketing &Management, 5 (3) (2016), pp. 214-226,
[6] John L., Crompton, and Paul K Ankomah.Choice set propositions in destination decisions [J].Annals of Tourism Research, 20(4)(1993), pp. 461-476.
[7] Meltem Caber, Tahir Albayrak, Duane Crawford. Perceived value and its impact on travel outcomes in youth tourism. [J]. [Journal of Outdoor Recreation and Tourism](https://www.sciencedirect.com/journal/journal-of-outdoor-recreation-and-tourism), [31](https://www.sciencedirect.com/journal/journal-of-outdoor-recreation-and-tourism/vol/31/suppl/C)(2020),pp. 100327.
[8] Cronin J J,Brady M K,Hult G T M.Assessing the effects of quality,value,and customer satisfaction onconsumer behavioral intentions in service environments.[J]. Journal of Retailing, 2(2000).
[9] H. Çevik. The Relationship Between Park Satisfaction, Place Attachment and Revisit Intention in Neighborhood Parks with Physical Activity Facilities. [J]. Educ. Learn., 9 (2) (2020), pp. 178.
[10] Nikolaos Stylos, Victoria Bellou, Andreas Andronikidis, Chris A. Vassiliadis.Linking the dots among destination images,place attachment,and revisit intentions:a study among British and Russian tourists[J].Tourism Management, 60(1)(2017), pp. 15-29.
[11] Ryu K, Han H, Kim T H.The Relationships among Overall Quick-casual Restaurant Image, Perceived ValueCustomer Satisfaction, and Behavioral Intentions.[J].International Journal of Hospitality Management, 27(3) (2008), pp. 459-469.
[12] Fangging Lu. Museum architecture as spatial storytelling of historical time: Manifesting a primary example of Jewish space in Yad Vashem Holocaust History Museum. Frontiers of Architectural Research. 2017; 6 (4): 442-455. DOI: 10.1016/j.foar.2017.08.002
[13] S M A Moin,  Sameer Hosany,  Justin O'Brien. Storytelling in destination brands’ promotional videos. [Tourism Management Perspectives](https://www.sciencedirect.com/journal/tourism-management-perspectives). 2020; [34](https://www.sciencedirect.com/journal/tourism-management-perspectives/vol/34/suppl/C): 100639. DOI: 10.1016/j.tmp.2020.100639

**Lastly, we would like to express our sincere gratitude to the reviewers and editors for their constructive feedback and guidance on this paper. I wish you all the best in your work and hope that PLOS ONE will continue to thrive and prosper.**
